# Prevalence and root causes of surgical site infection among women undergoing caesarean section in Ethiopia: a systematic review and meta-analysis

**DOI:** 10.1186/s13037-019-0212-6

**Published:** 2019-10-28

**Authors:** Fentahun Adane, Abay Mulu, Girma Seyoum, Alemu Gebrie, Akilog Lake

**Affiliations:** 1 Department of Anatomy, College of Health Sciences, Addis Ababa University, Addis Ababa, Ethiopia; 2 Department of Biomedical Science, School of Medicine, Debre Markos University, Debre Markos, Ethiopia; 3 Department of Gynecology & Obstetrics, School of Medicine, Debre Markos University, Debre Markos, Ethiopia

**Keywords:** Caesarean section, Surgical site infection, Root causes, Ethiopia

## Abstract

**Background:**

Surgical site infection is a common complication in women undergoing Caesarean section and the second most common cause of maternal mortality in obstetrics. In Ethiopia, prevalence and root causes of surgical site infection post-Caesarean section are highly variable. This systematic review and meta-analysis estimate the overall prevalence of surgical site infection and its root causes among women undergoing Caesarean section in Ethiopia.

**Method:**

Systematic review and meta-analysis were conducted to assess the prevalence and root causes of surgical site infection in Ethiopia. The articles were searched from the databases such as Medline, Google Scholar and Science Direct. A total of 13 studies from different regions of Ethiopia reporting the prevalence and root causes of surgical site infection among women undergoing Caesarean section were included. A random effect meta-analysis model was computed to estimate the overall prevalence. In addition, the association between risk factor variables and surgical site infection related to Caesarean section were examined.

**Results:**

Thirteen studies in Ethiopia showed that the overall prevalence of surgical site infection among women undergoing Caesarean section was 8.81% (95% CI: 6.34–11.28). Prolonged labor, prolonged rupture of membrane, presence of anemia, presence of chorioamnionitis, presence of meconium, vertical skin incision, greater than 2 cm thickness of subcutaneous tissue, and general anesthesia were significantly associated with surgical site infection post-Caesarean section.

**Conclusion:**

Prevalence of surgical site infection among women undergoing Caesarean section was relatively higher in Ethiopians compared with the report of center of disease control guideline. Prolonged labor, prolonged rupture of membrane, presence of anemia, chorioamnionitis, presence of meconium, vertical skin incision, greater than 2 cm thickness of subcutaneous tissue and/or general anesthesia were significantly associated with surgical site infection post-Caesarean section.

## Background

Caesarean section (C/S) is a surgical practice where a neonate is born via an incision through both the abdominal wall and uterus. It is the most frequent surgical procedure in obstetrics, occurring in approximately 15% of all deliveries, but ranges from approximately 3.5% across the African continent to 29.2% in Latin America [1].

While surgery is an essential element in health care, infections and complications following surgery contribute to maternal morbidity and mortality. Surgical site infections (SSIs) are those infections which are confined to the incisions and/or involve structures adjacent to the wounds that were exposed during surgery [2]. A C/S related SSI was previously defined operationally as an infection involving the abdominal incision or the uterus [3, 4]; however, more recently it has expanded to any infection occurring within 30 days post-surgery involving either the incision or deep tissue at the site [5, 6].

SSIs in obstetrics is the second most common cause of maternal mortality next to postpartum hemorrhage [7]. These events are one of the most common nosocomial infections accounting for 14–16% of the inpatient infections [8] and 20–25% of all hospital-acquired infections worldwide [9]. Women undergoing Caesarean deliveries have a 5 to 20-fold greater chance of getting an infection compared with women who give birth vaginally. These SSIs post-Caesarean birth can occur in the pelvic organs, around the surgical incision, and sometimes the uterine wall [10]. In addition, maternal morbidity related to infections post-C/S is eight times higher than post-vaginal delivery [11].

In Ethiopia, previous studies reported a 14.8–59% prevalence of hospital acquired infections [12–15];and SSIs were indicated as the most likely cause of nosocomial infection in obstetrics and gynecology than in general surgical wards [16].

Despite improvements in operating room practices, instrument sterilization methods, better surgical technique, and the best efforts of infection prevention strategies, SSIs remain a major cause of hospital-acquired infections and rates are increasing globally even in hospitals with modern facilities and standard protocols of preoperative preparation and antibiotic prophylaxis [17]. Worldwide, SSIs have been responsible for the growing cost; morbidity and mortality correlated with surgeries, and continued to be difficult to address [18].

Although antibiotics are available, SSIs are still responsible for much morbidity and far reaching socioeconomic consequences especially in developing countries like Ethiopia. A better understanding of magnitude and causes might improve SSIs infection control.

The overall prevalence and causes of SSI among women undergoing C/S in Ethiopia have not yet been investigated. Therefore, the main aim of this systematic review and meta-analysis was to estimate the pooled prevalence and to identify the root causes of SSI in Ethiopia. The findings of this meta-analysis will help policy makers and other concerned bodies in planning and implementing strategies to prevent and/or mitigate impacts of SSIs. The study could also be used as a baseline for researchers to carry out investigations on related topics. The review question is: What are the prevalence and associated risk factors for SSI among women undergoing C/S in Ethiopia?

## Methods

### Identification and study selection

Both peer reviewed published articles and unpublished research reporting the prevalence and root causes of SSI among women undergoing C/S in Ethiopia were searched by three authors (FA, AM, and AG). Eligible studies in English language only were identified through a literature search of Medline (PubMed), EMBASE, HINARI, Google Scholar, Science Direct, Cochrane Library, and other sources. The references of each included article were also searched manually to exhaustively retrieve the articles. The search of the articles was performed from January 9, 2019, until March 15, 2019. Unpublished studies such as theses/dissertation and reports from digital library catalogues were also searched through Google and Google Scholar. The terms for the search were pre-defined for a comprehensive search strategy that included all fields within records, and Medical Subject Headings (MeSH terms) for expanding the search in an advanced PubMed search. In the Boolean operator, within each axis, we combined keywords with the “OR” operator and we then linked the search strategies for the two axes with the “AND” operator. The search terms used for the search were “Prevalence” OR “Epidemiology” AND “Caesarean” AND/OR “Caesarean section” OR “Cesarean” AND/OR “Cesarean section” AND “SSI” AND/OR “Surgical site infection” AND/OR “Incision Infection” AND “Obstetric” AND/OR “Obstetrics” AND/OR “Obstetrical” AND” Mothers “OR “Maternal” AND/OR “Women” AND/OR “Females” AND” Ethiopia” AND/OR “Ethiopian hospitals”. The specific searching detail in pubmed with MeSH terms was (“surgical wound infection”[MeSH Terms] OR (“surgical”[All Fields] AND “wound”[All Fields] AND “infection”[All Fields]) OR “surgical wound infection”[All Fields] OR (“surgical”[All Fields] AND “site”[All Fields] AND “infection”[All Fields]) OR “surgical site infection”[All Fields]) AND (“Caesarean section”[All Fields] OR “Cesarean section”[MeSH Terms] OR (“Cesarean”[All Fields] AND “section”[All Fields]) OR “Cesarean section”[All Fields]) AND (“ethiopia”[MeSH Terms] OR “ethiopia”[All Fields]). All the literatures accessible until March 15, 2019 were included in the present study. The systematic review and meta-analysis was guided with the Preferred Reporting Items for Systematic Reviews and Meta-Analyses (PRISMA) guidelines [19].

### Eligibility criteria

#### Inclusion criteria

Studies reporting prevalence and causes of SSI among women undergoing C/S in Ethiopia were included.

##### Study area

Only articles conducted in or including Ethiopian hospitals.

##### Study design

All observational studies (cross-sectional, case controls and cohort) that contain original (primary) data reporting of the prevalence and associated risk factors SSI among women undergoing C/S in Ethiopia were considered.

##### Language

Literature published in the English language only was included.

##### Population

Studies conducted among women undergoing C/S were considered.

##### Publication condition

Both published articles and unpublished studies were considered.

#### Exclusion criteria

Non-accessible research whether published or unpublished if irretrievable from the internet or failures of reply to correspondences made by e-mail to the corresponding author within two weeks were excluded. Besides, research which did not report our outcome of interest, were excluded after reviewing (by three authors, FA, GS and AL) per our protocol described herein.

### Data abstraction

All the necessary data were retrieved using a consistent data extraction format in Microsoft Excel™ by two authors (FA and AG). For the prevalence of SSI, the data extraction format included first author, the region where the study was conducted, and the hospital where the study was carried out, publication year, study design, sample size, and prevalence of SSI.

For the root causes, the data extraction format was prepared for each specific potential cause (i.e., rupture of membrane more than 12 h, chorioamnionitis, prolonged labor, meconium, anemia, type of incision, and type of anesthesia). The researchers chose these variables because they are the most commonly reported associated risk factors in the studies included in this meta-analysis. In this systematic review and meta-analysis, the investigator considered variables as root causes if two or more studies mentioned them as risk factors (cause). For every root cause, to compute the odds ratio, the data from the primary studies were extracted in the form of two by two tables by the two authors (FA and AG).

### Outcome measurements

This systematic review and meta-analysis has two major outcomes. The primary outcome is to determine prevalence of SSI among women undergoing C/S in Ethiopia. The overall prevalence was calculated by dividing the number of mothers who develops SSI to the total number of mothers who delivered by C/S in the study period who have been included in the study (sample size) multiplied by 100. The second outcome of the study was to identify the root causes of surgical site infection.

### Quality assessment

To evaluate the quality of the studies included in this review, two researchers (FA and AM) applied the Newcastle-Ottawa Scale tool as modified for cross-sectional studies’ quality assessment [20]. The tool consists of three major parts. The first part has potentially five stars and assesses the methodological quality of each study with one star indicating poor quality and five stars indicating excellent quality. The second part of the tool assesses the comparability of the studies with up to two stars to be assessed. The last part determines the quality of the original articles with respect to their statistical analysis and outcomes with a possibility of up to three stars. Using the tool as a checklist, the qualities of each of the original articles were evaluated independently by the two authors. Any difference between the authors on quality assessment result was solved by discussion. Articles with medium to high (at least three of five stars on the first part of the tool) and high quality (≥6out of 10 across the three parts) were included for the analysis.

### Statistical analysis

The required data were collected using a Microsoft Excel™ form and analyzed by using STATA Version 15.0 software. The original articles were presented using tables and forest plot. The researcher calculated the standard error of prevalence for each original article by the binomial distribution formula. Heterogeneity among the reported prevalence of studies was checked by using heterogeneity χ2 test, I^2^ test and the *p*-values [21]. The above statistical tests indicated that there was a significant heterogeneity among the studies (I^2^ = 95.5%, *p <* 0.000). As a result, a random effects meta-analysis model was applied to estimate the Der Simonian and Laird’s pooled effect. In addition, univariate meta-regression model was conducted by taking publication year and sample size of the studies to assess the probable source of heterogeneity, but none was statistically significant. Possible publication bias was also evaluated objectively by using Egger’s correlation and Begg’s regression intercept tests at 5% significant level respectively [20, 22]. The Egger’s weighted regression and Begg’s rank correlation test methods were also used to assess publication bias and was insignificant (*P* < 0.059). Furthermore, to reduce the random discrepancies between the point estimates of the primary study, sub-group analysis was carried out based on region of studies.

## Results

### Search results

**Fig. 1:**
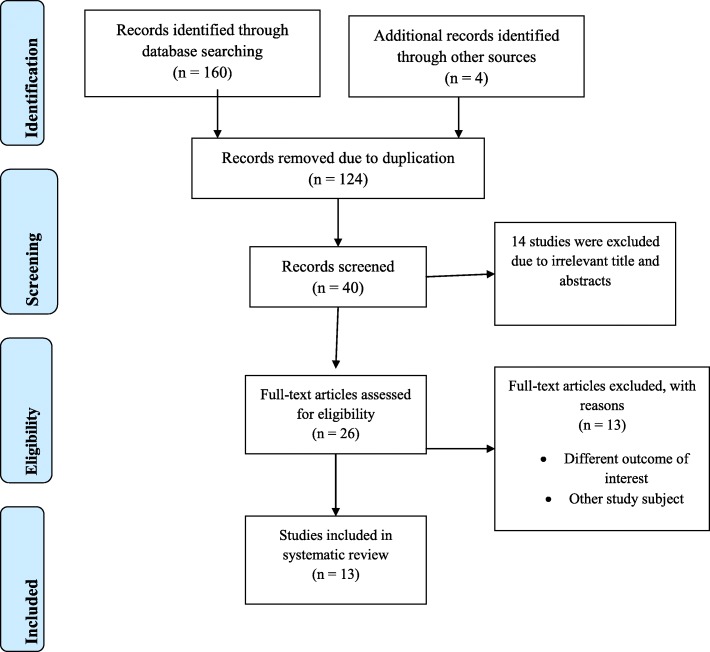
Flow chart describing the selection of studies for the systematic review and meta-analysis of prevalence and root causes of surgical site infection among women undergo cesarean section in Ethiopia, (showing how articles were identified, screened, and eligible ones were included for the studies)

A total of 164 articles regarding prevalence and root causes of surgical site infection in Ethiopia were retrieved from the databases of Medline (PubMed), EMBASE, HINARI, Google Scholar, Science Direct, Cochrane Library and other sources described above. From these preliminary records, 124 articles were excluded due to duplication. From the remaining 40 articles, 14 articles were excluded as they were found to be not eligible to this review after assessing their titles and abstracts. The remaining 26 full text articles were then accessed, and assessed for eligibility based on the preset criteria, which resulted in further exclusion of 13 articles primarily due to the study population and/or outcome of interest. Among these, four of the studies were conducted in countries other than Ethiopia: Nigeria [23], China [24], India [25] and England [26]. The remaining nine studies were conducted in different regions of Ethiopia [27–35] and excluded because of the study population and unreported outcome of interest. Finally, 13 eligible studies were included in the review (Fig. 1).

### Characteristics of original articles

A total of 13 original studies that reported the prevalence of SSI among women undergoing C/S and its root causes were included in this systematic review and meta-analysis. The studies were conducted from 2010 to 2019. The study design for all included research was cross-sectional. In this study, 8496 study participants were included to estimate the pooled prevalence of SSI and its root causes in Ethiopia. The quality score of the research ranged from 6 to 8 out of 10 as assessed by the Newcastle-Ottawa tool described previously. The studies were conducted in Oromia Region [36–38], Southern Nations and Nationalities of Peoples (SNNP) Region [39–41], Tigray Region [42–44], Amhara Region [45, 46] and Addis Ababa [47, 48]. The sample size ranged from 98 in Oromia Region [37] to 2911 Tigray Region [43] (Table 1).

**Table 1:** Descriptive summary of 13 studies reporting the prevalence of SSI among women undergoing Caesarean section in Ethiopia included in the systematic review and meta-analysis

Author	Publication year	Region	Study Hospital	Sample Size	Case	Prevalence 95% CI
Amenu, Belachew et al. [36]	2011	Oromia	Jimma University Specialized Hospital	770	88	11.40 (9.16, 13.64)
Bizuneh and Ayana [47]	2018	Addis Ababa	St Paul’s Hospital Medical College	582	17	2.90 (1.54,4.26)
Dacho and Angelo [39]	2018	SNNP	Mizan Tepi University Teaching Hospital	325	42	12.90 (9.26, 16.54)
Fantu, Segni et al. [37]	2010	Oromia	Jimma University Specialized Hospital	98	35	35.70 (26.21,45.19)
Gedefaw, Asires et al. [45]	2018	Amhara	Felegehiwot Referral Hospital	447	42	9.40 (6.48, 12.32)
Gelaw and Abdela [48]	2018	Addis Ababa	Zewditu Memorial Hospital	563	40	7.10 (4.98, 9.22)
Gelaw, Aweke et al. [42]	2017	Tigray	Lemlem Karl Hospital	384	26	6.80 (4.28,9.32)
Mamo, Abebe et al. [38]	2017	Oromia	Assela Teaching Referral Hospital	384	36	9.40 (6.48, 12.32)
Rose, Fekad et al. [46]	2018	Amhara	Felege hiwot Referral Hospital	247	21	8.50 (5.02, 11.98)
Tadesse, Gessessew et al. [43]	2019	Tigray	Ayder Comprehensive Specialized Hospital	2911	49	1.70 (1.23, 2.17)
Tesfaye, Hailu et al. [40]	2017	SNNP	Yirgalem General Hospital	469	34	7.20 (4.86, 9.54)
Wendmagegn, Abera et al. [44]	2018	Tigray	Ayder Comprehensive Specialized Hospital	206	24	11.70 (7.31,16.09)
Wodajo, Belayneh et al. [41]	2017	SNNP	Hawassa University Teaching and Referral Hospital	1110	65	5.90 (4.51, 7.29)

### Meta-analysis

#### Prevalence of surgical site infection among women undergoing caesarean section in Ethiopia

**Fig. 2:**
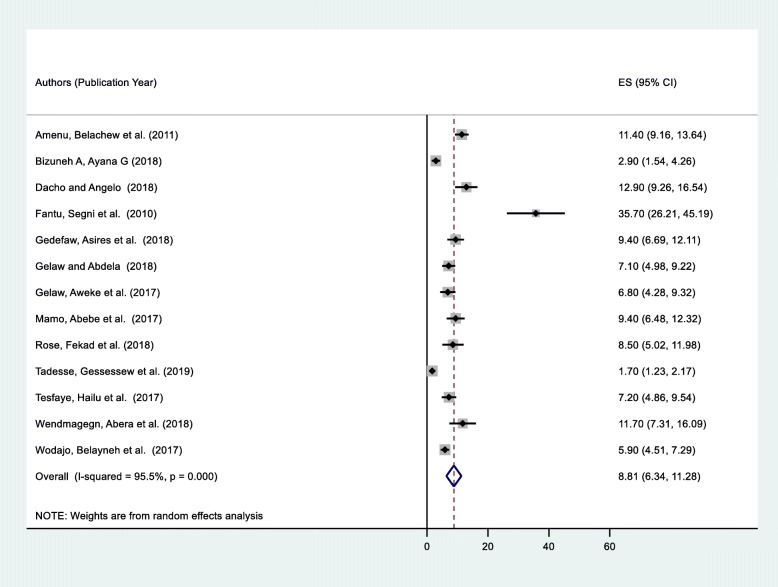
Forest plot of the pooled prevalence of SSI among women undergoing cesarean section in Ethiopia, 2019

Thirteen studies across 5 regions of Ethiopia yielded an overall prevalence of SSI 8.81% (95% CI: 6.34–11.28) among mothers who delivered by C/S (Fig. 2). Considerable heterogeneity was found across the studies as revealed by I^2^ statistic (I^2^ = 95.5, *p* value *<* 0.05); hence, a random effect model was used to estimate the pooled prevalence of SSI post-C/S in Ethiopia. A univariate meta-regression model was also carried out to identify the possible sources of heterogeneity, by considering factors, such as publication year and sample size although none of these variables was found to be statistically significant. Beggs’ and Eggers’ tests also indicated the absence of statistically significant publication bias (*p* > 0.05 for both).

#### Sub-group analysis

**Fig. 3:**
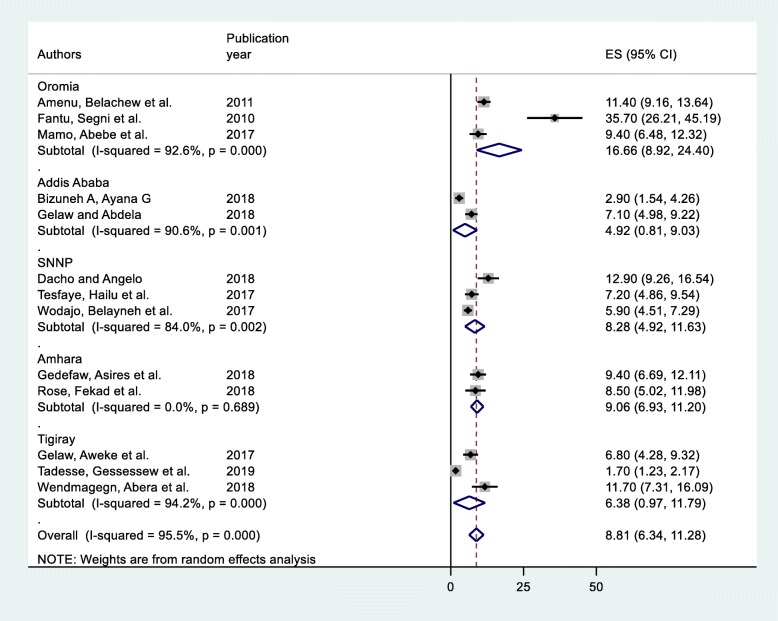
Forest plot of the Sub group analysis of prevalence of SSI among women undergoing cesarean section in Ethiopia, 2019

Due to considerable heterogeneity among the included articles in this study, sub-group analysis based on study region was considered to identify the possible source of heterogeneity across studies. The highest prevalence was observed in Oromia Region with a prevalence of 16.66 per 100(95% CI: 8.92, 24.40) followed by Amhara Region, 9.06 per 100 (95% CI: 6.93, 11.20);SNNP Region,8.28 per 100 (95% CI: 4.92, 11.63); Tigray Region, 6.38 per 100(95% CI: 0.97, 11.79) and Addis Ababa, 4.92 per 100 (95% CI: 0.81, 9.03) (Fig. 3).

#### Root causes of surgical site infection among women undergoing C/S in Ethiopia

Prolonged labor (≥25 h) (95% CI: 2.65–10.06), prolonged rupture of membrane (≥13 h) (95% CI: 3.33–8.29), presence of anemia (95% CI: 3.77–23.51), presence of chorioamnionitis (95% CI: 4.05–21.45), presence of meconium (95% CI: 1.41–9.99), vertical skin incision (95%CI 2.61–5.85), greater than 2 cm thickness of subcutaneous tissue (95%CI: 0.83–5.92 and general anesthesia (95%CI: 2.02–5.85) were significantly associated with surgical site infection.

Mothers with prolonged labor (25 or more hours) at the time of the C/S had 5.16(95% CI: 2.65–10.06) times increased odds of SSI than their counterparts. The odds of SSI were also increased by 5.26(95% CI: 3.33–8.29) among mothers who had prolonged rupture of membrane (rupture of membranes for 13 or more hours). Mothers who had anemia, chorioamnionitis and/or meconium were9.41 (95% CI: 3.77–23.51), 9.32(95% CI: 4.05–21.45) and 3.76 (95% CI: 1.41–9.99) times more likely at high risk for SSIs as compared to those mothers with none of these conditions prior to C/S, respectively.

Study subjects who underwent vertical skin incision were 3.91(95%CI: 2.61–5.85) times more likely to develop SSIs than those who had transverse skin incision. Mothers whose subcutaneous tissue thickness was greater than 2 cm were 2.82 (95%CI: 1.61–4.94) times more likely at risk for SSI than mothers whose subcutaneous tissue thickness was less than 2 cm.

Finally, mothers who had C/S by general anesthesia were 2.69(95%CI: 2.02–5.85) times more likely to develop SSI as compare with mothers who had C/S by spinal anesthesia (Table 2).

**Table 2:** Factors (root causes) of surgical site infection

Variables	OR	95% CI
Prolonged labor (≥25 h)	5.16	(2.65–10.06)
Prolonged rupture of membrane (≥12 h)	5.26	(3.33–8.29)
Maternal anemia	9.41	(3.77–23.51)
Presence chorioamnionitis	9.32	(4.05–21.45)
Presence meconium	3.76	(1.41–9.99)
Vertical skin incision	3.91	(2.61–5.85)
Subcutaneous tissue thickness > 2 cm	2.82	(1.61–4.94)
General anesthesia	2.69	(2.02–5.85)

## Discussion

The aim of this systematic review and meta-analysis was to estimate the pooled prevalence of SSI post-C/S and its root causes in Ethiopia. The study has found that the overall prevalence of SSI in Ethiopia among women undergoing C/S is 8.81%. This finding is similar to studies conducted in Cameroon [49] and Nigeria [23] which have reported SSIs prevalence of 9.16 and 9.1%, respectively. The prevalence of SSIs observed in the present study is slightly less than studies conducted in Tanzania and Nepal which reported prevalence of 10.9 and 12.6% respectively [50, 51]. However, this study has higher prevalence than reports from Turkey (0.3%), United States of America (5.2%), and Italy (1.6%) [18, 52, 53]. There could be numerous reasons for the heterogeneity of the prevalence rates among the various studies as compared with the present study. Higher prevalence rates of SSIs in developing countries may be due to limited hygienic practice unlike developed countries with relatively better standards of infection control policies and practices. Difference in population sampling, study design, and ethnicity may also contribute to the variation in the prevalence rates in the above studies as compared to the present study.

The sub-group analysis of this study demonstrated that the prevalence of SSI among women undergoing C/S significantly varies across regions. The highest prevalence of SSI was observed in Oromia Region, followed by Amhara, SNNP, and Tigray Regions, while the lowest prevalence was observed in the urban region of Addis Ababa. Possible justifications for this pattern could be population variation across the regions. According to Ethiopian Central Statistical Agency, in 2015, the total number of peoples were higher in Oromia followed by Amhara, SNNP, Tigray then Addis Ababa [54]. This population potentially leads to excessive numbers of mothers in obstetric wards with likely cases of infection [55]. Another possible reason could be due to poor dietary style, poor personal hygiene, and low socioeconomic capacity of rural people across regions than urban populations leading women to be vulnerable to nosocomial post-C/S SSIs.

The present study shows that there are significant associations between SSI and labor duration, rupture of membrane, anemia, chorioamnionitis, meconium, types of incision, thickness of subcutaneous tissue, and types of anesthesia. In this study, mothers with prolonged labor (≥25 h) and prolonged rupture of membrane (≥13 h) before operation had considerably increased risk of SSI than their counterparts. Studies carried out in Kenya [56], Nigeria [23], Qatar [57] and Israel [58] reported comparatively similar conclusions. Normally, during pregnancy, amniotic fluid and cervical mucus server as barriers to infection. However, if the membrane is ruptured, this protective effect is gradually reduced over time as amniotic fluid becomes no longer sterile. It, therefore, appears that prolonged labor and rupture of membranes contribute to amniotic fluid migration from the normal flora of the lower genital tract and direct to surgical site and peritoneal cavity [59].

In our study, anemia was found to be predicative of SSIs showing women with anemia having 9.41 times more likelihood to develop SSIs as compared to non-anemic women. This finding was in line with previous studies conducted in Nigeria [60], India [61], and China [59] and may reflect that low hemoglobin concentration reduces the oxygen tension in the wound site and increases the risk of SSI by compromising the activity of macrophages [49] and delaying infection healing progress [62].

In the current study, women with chorioamnionitis were 9.32 times more likely to develop SSIs when compared to those women who had no chorioamnionitis. Similar findings have been reported in Sub-Saharan African countries, India, and United States of America [63–65]. The potential explanation might be that unbroken membranes serve as a barrier to infections from the lower genital tract to the uterine cavity, which could also be due to iatrogenic infectivity of the peritoneum during surgery.

In the present study, women with meconium were 3.76 times more likely to develop SSIs when compared to those women who had no meconium. This finding is in line with a previous study which reported that occurrence of thick meconium in amniotic fluid is strongly associated with SSIs [66].

In addition, women who had vertical abdominal incisions were more likely to develop surgical site infections as compared to those women who had transverse incisions, which was also reflected in a study conducted in Nigeria [67]. The rationale may be due to the fact that a vertical incision is performed in the lower midline through the linea Alba which has poor blood supply. As a result, the area of incision may undergo necrosis and subsequent degeneration after incision if its edges are not aligned properly during closure.

Thickness of subcutaneous tissue more than 2 cm was also one of the causes for surgical site infection in this study. Similar findings were reported in an American study [68]. This risk factor could be due to reduced vascularity of the subcutaneous tissue, serous fluid collection, and hematoma formation in an overweight woman.

Finally, mothers for whom operations were under general anesthesia were more likely to have SSIs compared to mothers under spinal anesthesia. This finding is in agreement with the studies conducted in various African countries which indicated that C/S and use of general anesthesia increase the risk of accidental internal organs damage and internal bleeding because of uterine atony [69–71].

### Limitations of the study

All articles considered in this systematic review and meta-analysis were cross-sectional by design. As a result, temporal associations between the factors and the outcome variables cannot be established. Most of the studies included in this review had a relatively small sample size which may affect the final estimation. In addition, as this meta-analysis included available studies reported from a small number of hospitals in Ethiopia, there may be under-representation of the different regions in the country.

## Conclusion

The prevalence of SSI among women undergoing C/S was higher compared to the standard center of disease control (CDC) guidelines of SSI [72]. The highest prevalence of SSI within the Ethiopian studies reviewed was observed in Oromia Region followed by Amhara Region, SNNP Region and Tigray Region, while the least prevalence was observed in Addis Ababa. Labor duration, rupture of membrane, anemia, chorioamnionitis, meconium, types of skin incision, thickness of subcutaneous tissue and types of anesthesia were significantly associated with SSI post-C/S. Therefore, based on the findings, it is recommended that efforts should be made to ensure that prevention of prolonged labor through early intervention in cases where there is protracted progress of labor. In addition, minimizing early artificial rupture of membranes should be encouraged to decrease incidence of prolonged rupture of membranes. Furthermore, effective post-operative antibiotics should be given to patients undergoing C/S, especially those who are at risk of post-C/S infection (i.e. anemia, chorioamnionitis, presence of meconium and obesity). Transverse incision and spinal anesthesia are recommended to minimize the incidence of SSI post-C/S. Finally, given the high prevalence of SSIs shown in this review, it is recommended that active SSI surveillance and infection prevention strategies be established nationally.

## Data Availability

The datasets used and/or analyzed during the current study are available from the corresponding author on reasonable request.

## Abbreviations

C/S: Caesarean section
CDC: Center of Disease Control
SNNP: Southern Nations and Nationalities of Peoples
SSI: Surgical site infection
